# The effects of adapted physical activity on physical activity levels and social adaptive behaviors in children with autism spectrum disorders: a study using the ICF-CY framework

**DOI:** 10.3389/fpsyt.2025.1708901

**Published:** 2025-12-11

**Authors:** Xiaomei Zhan, Ziwei Kuang, Xiafang Li, Yuqing Wang, Chunlian Yuan, Tonglin Shi, Yufei Zeng, Kai Cheng

**Affiliations:** 1 Autism Sports Intervention Centre, College of Physical Education, Jiangxi Normal University, Nan Chang, Jiangxi, China; 2 College of Physical Education, Nanchang Institute of technology, Nan Chang, Jiangxi, China; 3 Ji’an No.1 High School, Ji´an, Jiangxi, China

**Keywords:** adapted physical activity, ICF-CY, autism spectrum disorders, physical activity levels, social adaptive behaviors

## Abstract

**Objective:**

This study investigated the effects of an adapted physical activity (APA) program, based on the ICF-CY framework, on physical activity and social adaptive behaviors in children with autism spectrum disorder (ASD).

**Methods:**

41 children with ASD (aged 6~12) were ultimately included and divided into an experimental group (EG) and a control group (CG). The EG received the APA intervention, while the CG participated in routine extracurricular physical activity sessions. Both groups received 24-week interventions, with 3 sessions per week, each lasting 80 minutes. Assessments were conducted before and after the intervention using the ActiGraph wGT3X-BT triaxial accelerometer and the Child Adaptive Behavior Rating Scale (CABRS).

**Results:**

After 24 weeks, the EG showed significant improvements in adaptive behaviors (independent, cognitive, social/self-control, and total scores), reduced sedentary time, and increased light and moderate-to-vigorous physical activity compared to the CG.

**Conclusions:**

The ICF-CY-based APA program effectively enhanced physical activity levels and social adaptive behaviors in children with ASD.

## 1 Introduction

The International Classification of Functioning, Disability and Health-Children and Youth Version (ICF-CY), published by the WHO, is the first standardized framework in the world integrating health and functioning, disability, and social environmental factors (1). This framework focuses not only on the disease or disability itself, but more on the functional changes and abnormalities experienced by individuals on the physical, psychological, and social levels (2). It enables the systematic description of the interactions among body functions and structures, activities and participation, and environmental factors, thereby facilitating the precise identification of individual rehabilitation needs and the formulation of personalized intervention goals (3). The ICF-CY framework emphasizes the developmental dynamics of children, with a particular focus on their activity and participation in daily contexts. This enables a more comprehensive and systematic assessment of functional impairments in children with autism spectrum disorder (ASD) (4). Its integration into clinical practice offers a holistic perspective for functional diagnosis, rehabilitation assessment, and the development of intervention strategies for this population.

According to the recent studies, the global prevalence of ASD ranges from approximately 1% ~2% (5). In the United States, the prevalence rate stands at 32.2 per thousand (meaning 1 in every 31 children aged 8 years old) (6).In China, the prevalence of ASD reaches 1%, with children aged 6 to 12 years old showing a prevalence rate of 0.7% (7). ASD is a pervasive neurodevelopmental disorder with core symptoms primarily characterized of social interaction difficulties, repetitive and stereotyped behaviors, and restricted interests (8). Individuals with ASD often present with comorbid motor coordination deficits, sensory processing abnormalities, and impaired environmental adaptation (9). They typically exhibit marked limitations in cognition, physical activity, and adaptive functioning (10). Children with ASD engage in an average of only approximately 30 minutes of moderate-to-vigorous physical activity (MVPA) per day, alongside prolonged sedentary behavior (SB), both falling short of the recommendations set by the World Health Organization (11). This pattern of low activity participation may further compromise their social adaptive functioning through a cascade effect involving physical-cognitive-social interplays (12). Deficits in social adaptive functioning significantly diminish children’s opportunities for and quality of participation in social life, thereby hindering their ability to acquire essential social support and resources (13).These impairments, which typically manifest as difficulties in language communication, challenges in environmental adaptation, and limited self-care skills, not only restrict individuals’ psychological and physical development and independence in daily living but also increase the caregiving burden on families and society (14).

Currently, the primary therapies for children with ASD include medication, behavioral intervention, and adaptive physical activity (APA) (15). APA is an interdisciplinary practice designed to enable individuals with physical and intellectual disabilities to participate equitably in physical activities through systematic adjustments of rules, equipment, methodologies, and environments based on individual abilities and contextual needs (16). These activities include programs such as the Special Olympics, modified sports games, physical conditioning, and therapeutic exercise, all tailored for individuals with special needs. They are designed to promote recovery of body and cognitive functions and enhance overall adaptive capacity through adapted training modalities (17). APA is recognized as a cost-effective rehabilitative intervention for children with ASD (18). By enhancing children’s self-efficacy and experiences of active participation, it promotes the voluntary application of learned skills in daily life, increases overall physical activity levels (19), and improves physical, cognitive, social functioning in children with ASD (20). Most existing studies on APA interventions focus on a single activity format, typically targeting isolated or multiple motor skills or functional deficits. This approach suffers from fragmented intervention designs, inconsistent evaluation standards, and insufficient cross-disciplinary collaboration. Guided by the ICF-CY framework, this study conducted a comprehensive assessment of functional characteristics in children with ASD and developed an integrated, personalized, and dynamically adaptive APA program. Tailored to their interests, abilities, and individual functional needs, the program incorporates environment-specific strategies and aims to promote holistic improvements in physical activity and social participation. Based on this, the present study aims to evaluate the effects of an APA intervention on daily physical activity levels and social adaptive behaviors in children with autism, guided by the ICF-CY framework. The following hypotheses are proposed (1): The integrated APA intervention based on the ICF−CY framework will effectively increase daily physical activity levels and improve social adaptive behaviors in children with ASD (2). This structured and adaptable APA intervention model may provide a new pathway for innovating rehabilitation approaches for children with ASD.

## 2 Materials and methods

### 2.1 Participants

This study used G*Power 3.1.9.7 for sample size estimation. Based on a previous relevant study (21), a medium effect size (Cohen’s d = 0.65) was set, with the assumptions of homogeneity of variance and normal distribution. Using a two-sided significance level of α = 0.05 and a statistical power of 90% (Power = 0.90, β = 0.10), the minimum sample size calculated by the software was 32 subjects. To account for potential participant dropout during the study, a dropout rate of 20% was assumed, resulting in a final required sample size of no less than 40 subjects.

This study recruited 45 male children with ASD (aged 6~12 years) in Nanchang, China, through a We chat public platform. Inclusion Criteria (1): proof of diagnosis of ASD provided by a tertiary care hospital (2), intelligence quotient score between 35~69 (22) (3), capable of completing physical activity interventions and assessment tasks. Exclusion Criteria (1): presence of other chronic diseases or disabilities (2), participation in any structured physical activity program within the past six months (3), contraindications to physical exercise (e.g., severe heart disease) (4), use of medication that may interfere with study outcomes. During the experiment, four participants withdrew due to either attending less than 90% of the sessions or for other reasons, resulting in a final sample of 41 participants included in the data analysis. All personal data of participants were kept strictly confidential. The study was approved by the Ethics Committee of Jiangxi Normal University (IRB-JXNU-PEC-2024007) and conducted in accordance with the Declaration of Helsinki. Written informed consent was obtained from all parents/guardians prior to participation.

### 2.2 Study design

A single-blind, block randomization method was employed. All 45 participants were assigned numbers (1, 2, 3, … 45) and entered into an Excel spreadsheet. Using the RAND () function, 45 random numbers were generated and sorted in ascending order. The first 23 participants were assigned to the experimental group, and the remaining 22 to the control group. The allocation was concealed using sealed envelopes (23). To prevent bias, assessors were blinded throughout the entire process, including participant recruitment, random sequence generation, and group assignment. Independent samples t-tests showed no statistically significant differences in the mean age, height, or weight between the experimental and control groups (P > 0.05). A 2 (group: experimental, control) × 2 (time: pre-test, post-test) mixed-design experiment was employed, with group as the between-subjects factor and time as the within-subjects factor. The experiment consisted of three phases: pre-intervention assessment, intervention, and post-intervention assessment. During the intervention period, all participants attended the school’s regular physical education classes. The EG received the APA intervention, while the CG participated in routine extracurricular physical activity sessions. The intervention content for both groups primarily included fundamental motor skills, fine motor skills, sensory integration training, as well as group cooperative games and competitions. The intervention of both groups lasted for 24 weeks, with a frequency of 3 sessions per week, and each session lasting 80 minutes. All children were required to attend at least 90% of the sessions.

The ICF-CY assessments were conducted by two researchers experienced in autism rehabilitation and evaluation, both trained in using the ICF-CY checklist and qualifiers. The assessors were blinded to the study design and instructed not to inquire about any participant information. The assessment covered three domains: body functions, activities and participation, and environmental factors. It was based on the ICF-CY core sets (brief version for 6~16 years) (24), developed specifically for children and adolescents with autism. All assessments were administered at the autism sports intervention centre before the intervention (week 0) and after its completion (week 25). The full assessment process for each child and their parents required approximately 45~60 minutes.

#### 2.2.1 APA program using the ICF-CY framework

**Figure 1:**
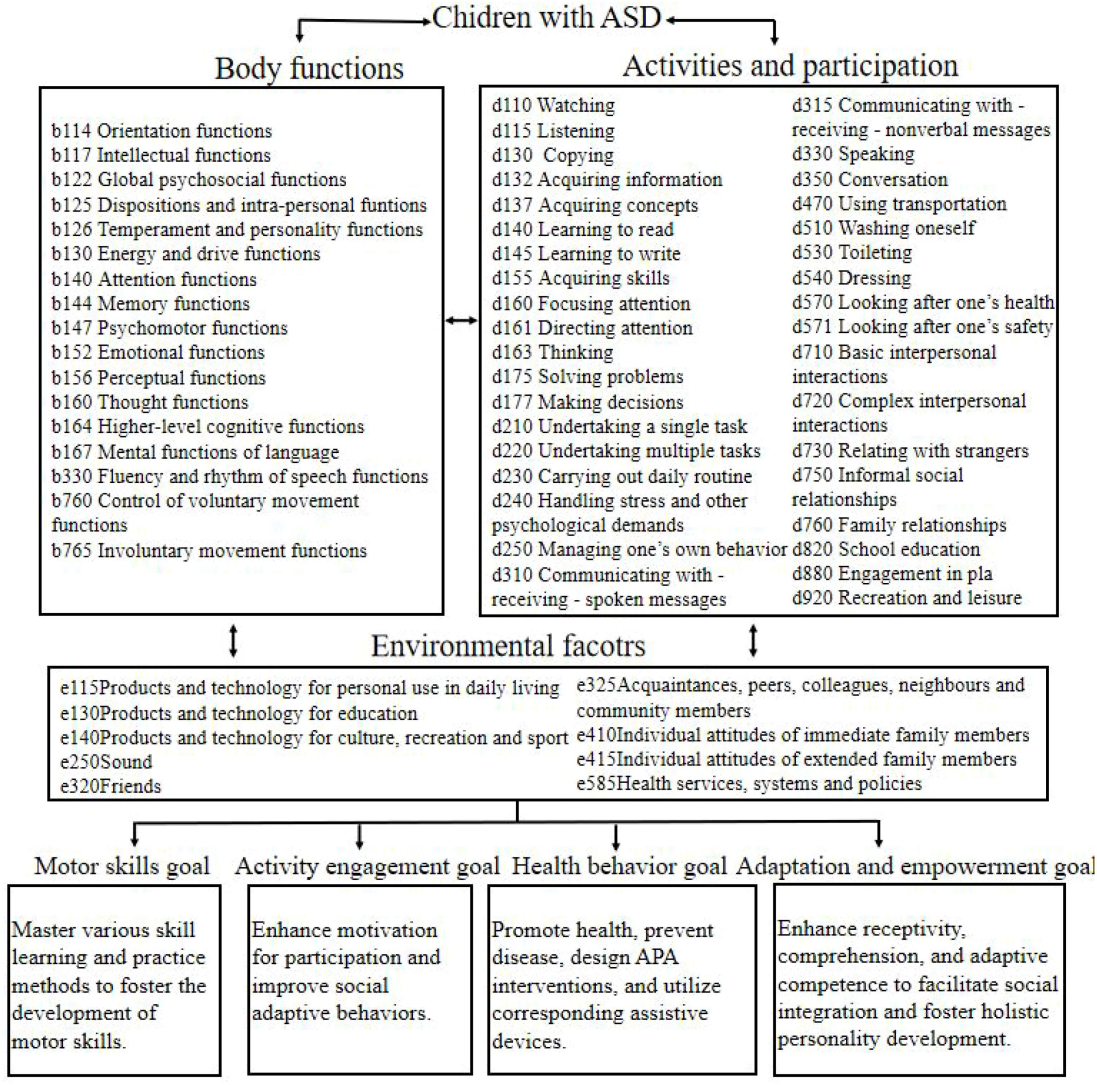
Tailored objectives of APA intervention for children with ASD based on the ICF-CY.

The ICF-CY is a health and functioning assessment framework specifically designed for individuals aged 0~18 years (2). The *Adapted Physical Education National Standards (Third Edition)* (25). serves as a key resource for understanding and delivering high-quality adapted physical education, emphasizing the evaluation of individuals with physical and intellectual disabilities. It comprehensively incorporates the most current standards and offers up-to-date knowledge and best practices for APA instruction. APA programs for children with ASD generally emphasize principles such as individualization, diversity, practical and life-oriented application, and repetitive practice. By adopting the ICF-CY’s terminology and coding system and integrating the pedagogical principles of APENS-3, corresponding APA intervention goals aligned with ICF-CY domains were designed (see **Figure 1**). Based on functional assessment results and these intervention goals, and taking into account the physical, motor, and psychological development levels of the children, an individualized APA intervention program was developed tailored to each participant’s characteristics (see **Table 1** for details).

**Table 1:** APA program for children with ASD based on ICF-CY.

ICF-CY categories	APA program components	ICF-CY codes
Body functions	1) Fundamental motor skill training Prone position, independent sitting, crawling, standing (double/single leg), walking (with natural arm swing), ascending/descending stairs, stepping over obstacles, kicking ball/beanbag), running, jumping (double/single leg, over obstacles), pedaling (tricycle/scooter board), etc. 2) Fine motor skill training Bead threading against time, block building, snowflake interlocking plates, pegboard activities, finger exercises, target throwing, equipment retrieval and organization, etc. 3) Sensory integration training Balance beam walking, tactile board massage, prone ball pushing, scooter board crawling, trampoline jumping, rolling exercises, Swiss ball activities. etc.	b114, b117, b122, b125, b126, b130, b140, b147, b152, b156, b160, b164, b167, b330, b760, b765
Activities and participation	1) Cooperative group games: ‘rainbow pass relay’, ‘blindfolded basketball’, ‘tunnel relay’, ‘three-legged race’, etc. 2) Social role-playing games: ‘animal Olympics’, ‘traffic commander’, ‘firefighter rescue’, ‘little train line-up’, etc. 3) Team competitions: ‘parent-child three-legged run’, movement chain relay’, ‘rainbow parachute tunnel crawl’, etc.	d110, d115, d130, d132, d137, d155, d160, d163, d175, d210, d230, d240, d250, d310, d315, d330, d350, d510, d530, d540, d570, d710, d750, d760, d820, d880, d920
Environmental factors	1) Safety measures: non-slip flooring, soft protective railings, etc. 1) Visual supports: star-track path design, step-by-step visual cue cards, etc. 2) Staff allocation: 1:1 staff-to-child ratio, pre-service volunteer training.	e115, e130, e140, e250, e320, e325, e410, e415, e585

#### 2.2.2 Adapting the content of APA program

Children with ASD exhibit significant heterogeneity, necessitating that APA program be individually tailored based on program objectives, individual characteristics, and practical constraints. Program implementation is a dynamic process, in practice, adjustments and optimizations must be continuously made in content selection, difficulty grading, and instructional strategies according to the child’s level of acceptance, learning progress, and resource utilization. These adaptations are essential to ensure effective participation in physical activities and to achieve the desired intervention outcomes (see **Table 2**).

**Table 2:** Adaptive adjustment of the APA program.

Adjustment dimension	Theoretical basis	Adjustment principles	Implementation strategies	Timing of adjustments/basis for judgment
Content pacing adjustment	Theory of motor Skill development	1) Skill progression pathway: fundamental motor skills → transitional skills → specialized skills. 2) Progression principle: strict adherence to mastery-based advancement.	1) Individualized pacing: dynamically adjusted based on skill acquisition. 2) Training emphasis: phased reduction of foundational drills with increased advanced skill practice.	1) Assessment metric: stage-specific skill mastery rate. 2) Monitoring focus: individual differences in learning pace.
Content difficulty adjustment	Zone of proximal development	1) Task difficulty must fall between the student’s current and potential ability levels. 2) Dynamically align challenge with developmental readiness to avoid over- or under-challenging tasks.	Difficulty progression: 1) Foundation phase: walking, running, jumping, and other fundamental skills. 2) Transition phase: introduce kicking, striking, throwing, and other transitional skills. 3) Proficiency phase: incorporate specialized skills such as cycling, sport-specific techniques, and competitive activities. Difficulty reduction strategies: 1) Regress to a previous difficulty level if stage objectives are not met. 2) Break tasks into smaller steps with visual supports. 3) Provide guided assistance and optimize environmental setup.	1) Skill acquisition efficacy. 2) Observable frustration during practice.
Instructional strategy adaptation	Peer-mediated intervention Cooperative learning Paraprofessional support	1) Implement diversified support strategies. 2) Combine group instruction with individualized guidance.	Group instruction and individual Tutoring: 1) Collaboration between teachers and volunteers. 2) Itinerant guidance from special education experts. 3) Parental involvement in support activities.	1) Student engagement and acceptance levels. 2) Availability of instructional resources.

### 2.3 Outcome variables

#### 2.3.1 Assessment of physical activity levels

Physical activity levels were measured using the ActiGraph wGT3X-BT triaxial accelerometer. This device has been widely used in research involving children, adolescents, and special populations, with demonstrated reliability and validity of 0.87 (26). The monitor was worn on the right side of the waist. Participants were instructed to wear it at all times except during water-based activities and sleep. The accelerometers were initialized with a sampling rate of 30 Hz. When using a 60-second epoch, the recommended intensity cut-points were as follows: sedentary behavior (0~100 counts·min^-1^), light physical activity (101~2288 counts·min^-1^), and moderate-to-vigorous physical activity (≥2289 counts·min^-1^) (27, 28). The monitoring period lasted for 7 consecutive days, including both weekdays and weekends. A valid day was defined as a minimum wear time of 10 hours (29), and a valid monitoring period required any 4 consecutive valid days (30), including at least 3 valid weekdays and 1 valid weekend day. The devices were retrieved the day after each testing period (pre- and post-intervention), and data were downloaded using the ActiLife software (version 6.13.3).

#### 2.3.2 Assessment of social adaptive behaviors

The Children’s Adaptive Behavior Rating Scale-Urban Edition (CABRS), developed and revised by Shuqiao Yao and Yaoxian Gong from Hunan Medical University in China, was used to assess adaptive behaviors in children with ASD (aged 3~12) (31). This scale consists of 59 items grouped into 8 subscales: sensorimotor, self‐care, language development, personal orientation, social responsibility, time and spatial orientation, labor skills, and economic activity. These are further categorized into three functional domains: independent functioning (including sensorimotor, self-Care, labor Skills, and economic activity), cognitive functioning (including language development, time and spatial orientation), and social/self‐control functioning (including Personal orientation and social responsibility). The scale demonstrated excellent reliability and validity, with a test-retest reliability coefficient ranging from 0.96 to 0.99 and correlation coefficients for all subscales and the total score exceeding 0.93 (18).

### 2.4 Quality control

Prior to the formal testing, all testing personnel received uniform training. A dedicated technician inspected all accelerometers for damage and ensured they were fully charged and functional before use. During the testing process, the researchers explained the purpose of the study and instructed the children, their parents, and caregivers on the proper wearing of the accelerometers. Throughout after-school physical activity sessions, staff provided ongoing supervision and guidance. Students completed the parental support questionnaire with assistance from trained staff, while parents received written instructions to complete their portion independently. After data collection, all records were compiled and cross-checked. Participants with missing data were excluded from statistical analysis to ensure data integrity and reliability. During the intervention, exercise intensity was monitored using Polar V800 heart rate sensors, with target heart rates maintained at 60%~80% of HRmax under dedicated monitoring. Each session was led by one head coach assisted by one assistant coach and multiple volunteers. All staff members received special education training and maintained a 1:1 ratio with children to ensure safety and instructional consistency.

### 2.5 Statistical analysis

Data were analyzed using SPSS 27.0. Descriptive statistics were performed, and the normality of the data was confirmed by the Kolmogorov–Smirnov test. Results are presented as mean ± standard deviation (M ± SD). A repeated-measures analysis of variance (ANOVA) was used to examine the effects on children’s sedentary time, moderate-to-vigorous physical activity time, and social adaptive behavior scores. Effect sizes for main and interaction effects are reported as partial eta-squared (partial η²). If a significant interaction effect was found, simple effect analysis was conducted, followed by pairwise comparisons with Bonferroni adjustment, along with their 95% confidence intervals (95% CI). The criteria for interpreting effect sizes were with values of 0.01, 0.06, and 0.14 indicating small, medium, and large effects, respectively (32). The significance level was set at p < 0.05.

## 3 Results

### 3.1 Changes in physical activity levels of children with ASD pre- and post the intervention

**Figure 2:**
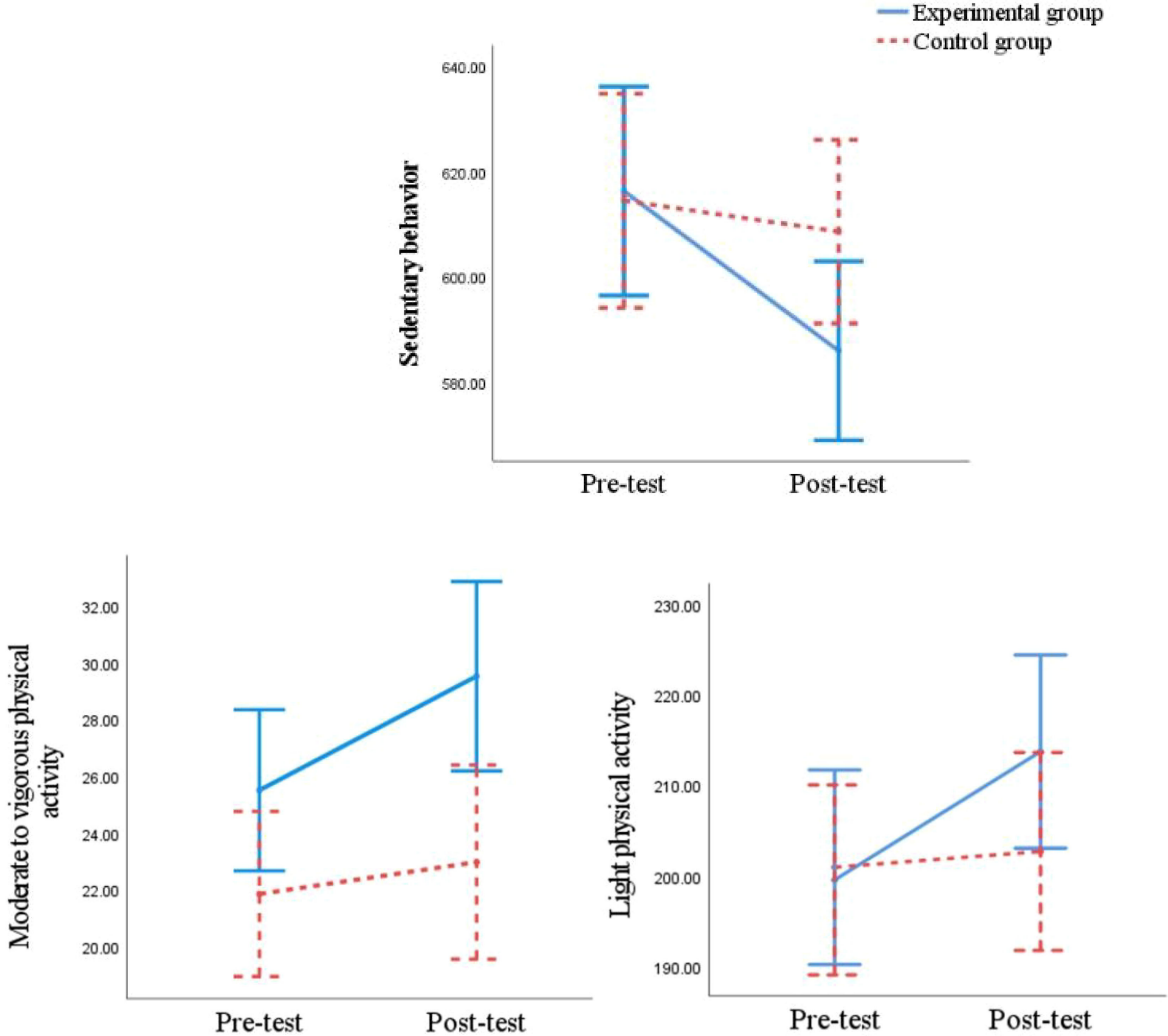
Analysis of changes in physical activity levels (min/day) in the experimental and control groups.

Repeated measures analysis of variance for physical activity levels (EG and CG) × time (pre-test and post-test) revealed (see **Table 3**; **Figure 2**). For SB time, a significant main effect of time was observed [F_(1, 39)_=17.269, *P* < 0.001, η*_p_*^2^ = 0.307]. The main effect of group was not significant [F_(1, 39)_=0.712, *P=*0.404>0.05, η*_p_*^2^ = 0.018], but a significant group × time interaction emerged [F_(1, 39)_=7.888, *P* = 0.008<0.01, η*_p_*^2^ = 0.168]. The *post-hoc* analyses further revealed that the EG showed a significant reduction in SB time from pre- to post-test (95% CI = 18.05 ~ 42.70, *p* < 0.001), whereas no significant change was observed in the CG (95% CI = -6.75 ~ 18.50, *p* = 0.352). Regarding LPA time, there was a significant main effect of time [F_(1, 39)_=8.786, *P* = 0.005<0.01, η*_p_*^2^ = 0.184], no significant main effect of group [F_(1, 39)_=0.474, *P* = 0.495>0.05, η*_p_*^2^ = 0.012], and a significant group × time interaction [F_(1, 39)_=5.354, *P* = 0.026<0.05, η*_p_*^2^ = 0.121]. The *post-hoc* analyses further revealed that the EG demonstrated a significant increase in LPA time after the intervention (95% CI = -21.74 ~ -6.58, *p* < 0.001), while the CG showed no significant change (95% CI = -9.51 ~ 6.02, *p* = 0.652). For MVPA time, significant main effects were found for both time [F_(1, 39)_=18.330, *P* < 0.001, η*_p_*^2^ = 0.320] and group [F_(1, 39)_=5.850, *P* = 0.020<0.05, η*_p_*^2^ = 0.130], along with a significant group × time interaction [F_(1, 39)_=5.824, *P* = 0.021<0.05, η*_p_*^2^ = 0.130]. The *post-hoc* analyses further revealed that a significant increase in MVPA time was found in the EG (95% CI = -5.71 ~ -2.32, *p* < 0.001), with no significant change in the CG (95% CI = -2.86 to 0.62, *p* = 0.199).

**Table 3:** Analysis of changes in physical activity levels (min/day) in the experimental and control groups.

Physical activity levels	EG (n=21)		CG (n=20)		F_(1, 39)_, *η_p_^2^*		
	Pre-test	Post-test	Pre-test	Post-test	Time	Group	Time×Group
SB	616.20 ± 43.73	585.83 ± 33.02	614.37 ± 46.33	608.50 ± 43.67	17.269, 0.307***	0.712, 0.018	7.888,0.168**
LPA	199.54 ± 20.08	213.70 ± 20.25	200.94 ± 27.06	202.68 ± 27.66	8.786,0.184**	0.474, 0.012	5.354,0.121*
MVPA	25.50 ± 6.79	29.51 ± 8.55	21.84 ± 6.04	22.96 ± 6.36	18.330,0.320***	5.850, 0.130*	5.824,0.130*

EG, experimental group, CG, control group; SB, Sedentary behavior, LPA, Light physical activity, MVPA, Moderate-to-vigorous physical activity; * P<0.05, ** P<0.01, ***P<0.001, the same below.

### 3.2 Changes in social adaptive behavior scores of children with ASD pre- and post the intervention

**Figure 3:**
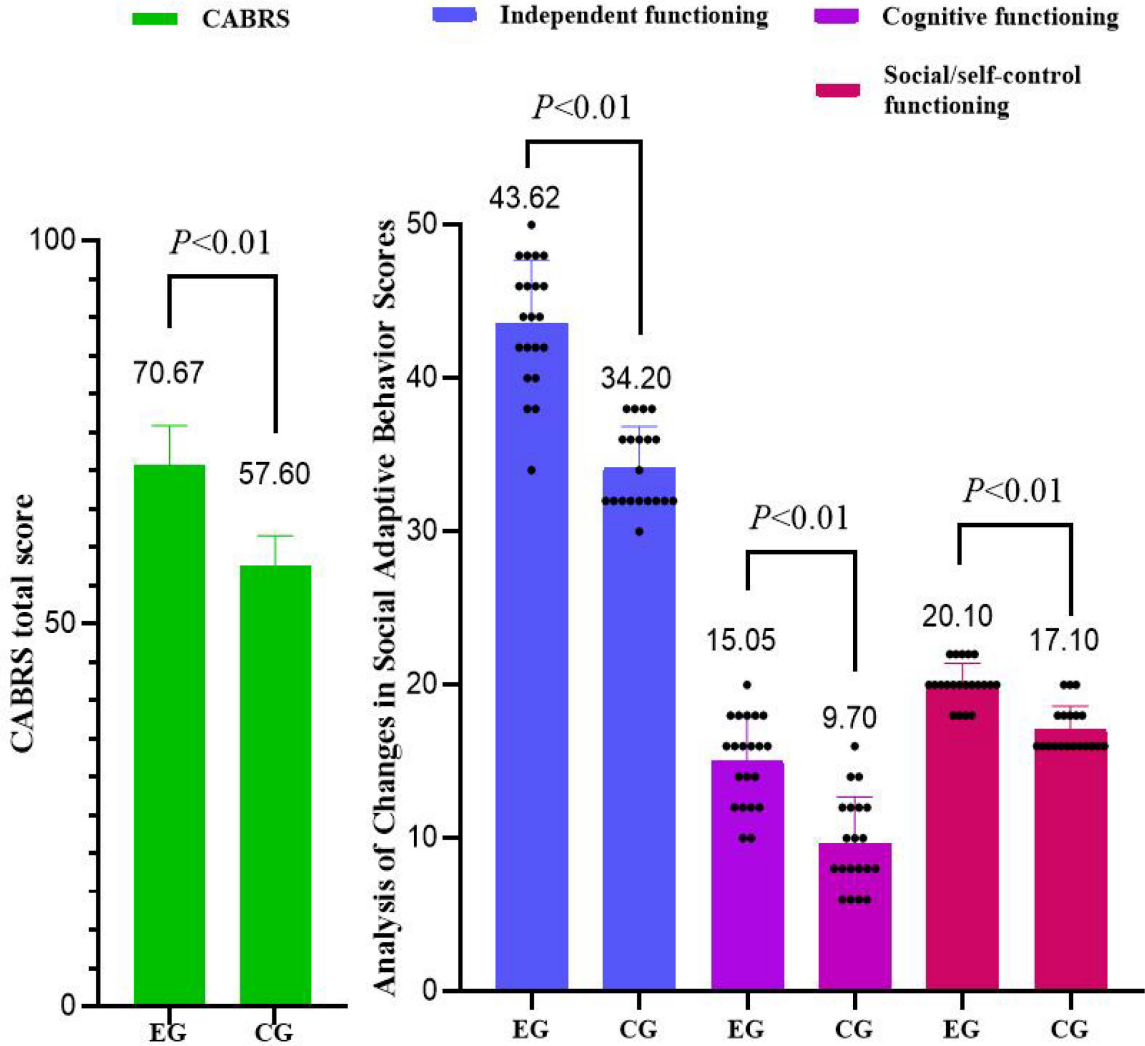
Analysis of changes in social adaptive behavior scores in the experimental and control groups.

Repeated measures analysis of variance for social adaptive behavior groups (experimental and CGs) × time (pre-test and post-test) revealed (see **Table 4**; **Figure 3**). For independent functioning, significant main effects were found for time [F_(1, 39)_=95.056, *P* < 0.001, η*_p_*^2^ = 0.709] and group [F_(1, 39)_=41.333, *P* < 0.001, η*_p_*^2^ = 0.515], along with a significant group × time interaction [F_(1, 39)_=47.516, *P* < 0.001, η*_p_*^2^ = 0.549]. The *post-hoc* analyses further revealed that the EG showed a significant increase in independent functioning scores from pre- to post-test (95% CI = -11.586 ~ -8.223, *p* < 0.001), while the CG exhibited no significant change (95% CI = -3.423 ~ 0.023, *p* = 0.053). In cognitive functioning, significant main effects emerged for time [F_(1, 39)_=18.424, *P* < 0.001, η*_p_*^2^ = 0.321] and group [F_(1, 39)_=16.649, *P* < 0.001, η*_p_*^2^ = 0.299], with a significant group × time interaction [F_(1, 39)_=15.939, *P* < 0.001, η*_p_*^2^ = 0.290]. The *post-hoc* analyses further revealed that a significant improvement in cognitive functioning scores was observed in the EG (95% CI = -7.408 ~ -3.640, *p* < 0.001), with no significant change in the CG (95% CI = -2.130 ~ 1.730, *p* = 0.835). For social/self-control functioning, significant main effects were observed for time [F_(1, 39)_=107.28, *P* < 0.001, η*_p_*^2^ = 0.735] and group [F_(1, 39)_=26.276, *P* < 0.001, η*_p_*^2^ = 0.403], as well as a significant group × time interaction [F_(1, 39)_=12.434, *P* = 0.001<0.01, η*_p_*^2^ = 0.242]. The *post-hoc* analyses further revealed that both the experimental and control groups demonstrated significant increases from pre- to post-test for social/self-control functioning (EG: 95% CI = -7.562 ~ -5.009, *p* < 0.001; CG: 95% CI = -4.408 ~ -1.792, *p* < 0.001). Although both groups showed significant improvements over time, the experimental group exhibited a greater magnitude of increase. Regarding the total scores, significant main effects were identified for time [F_(1, 39)_=226.489, *P* < 0.001, η*_p_*^2^ = 0.853] and group [F_(1, 39)_=53.726, *P* < 0.001, η*_p_*^2^ = 0.579], in addition to a significant group × time interaction [F_(1, 39)_=72.723, *P* < 0.001, η*_p_*^2^ = 0.651]. The *post-hoc* analyses further revealed that both groups showed significant improvements after the intervention in terms of total scores (EG: 95% CI = -19.837 ~ -15.591, *p* < 0.001; CG: 95% CI = -7.075 ~ -2.725, *p* < 0.001). The results indicated that while both groups changed significantly over time, the improvement in the experimental group was substantially greater than that in the control group.

**Table 4:** Analysis of changes in social adaptive behavior Scores in the experimental and control groups.

Social adaptive behavior	Experimental group (n=21)		Control group (n=20)		F_(1, 39)_, *η_p_^2^*		
	Pre-test	Post-test	Pre-test	Post-test	Time	Group	Time×Group
Independent functioning	33.71 ± 2.31	43.62 ± 4.08	32.50 ± 3.66	34.20 ± 2.67	95.056, 0.709***	41.333, 0.515***	47.516, 0.549***
Cognitive functioning	9.52 ± 3.28	15.05 ± 2.87	9.50 ± 2.82	9.70 ± 2.99	18.424, 0.321***	16.649, 0.299***	15.939, 0.290***
Social/self-control functioning	13.81 ± 1.54	20.10 ± 1.34	14.00 ± 2.25	17.10 ± 1.52	107.28, 0.735***	26.276, 0.403***	12.434, 0.242**
Total scores	52.95 ± 2.73	70.67 ± 5.15	52.70 ± 2.77	57.60 ± 3.87	226.489, 0.853***	53.726, 0.579***	72.723, 0.651***

## 4 Discussion

Regular and appropriate physical activity holds critical significance for children’s growth development and maintaining healthy lifestyles in adulthood. This study demonstrates that a 24-week APA intervention effectively reduced SB while increasing participation duration in both low-intensity and moderate-to-vigorous physical activities among children with ASD. Consistent with existing evidence, Firouzjah’s (33) study indicates that regular physical activity enhances activity engagement in ASD populations through improved sensory integration capabilities. Additionally, a meta-analysis corroborates the efficacy of structured exercise programs in reducing SB within this group (34). Compared to previous studies, a more significant increase in physical activity levels was observed among children with ASD. This may be attributed to the targeted nature of the intervention program, wherein activity content was tailored to individual children’s interests and fully integrated with visual supports, greatly enhancing participation motivation and feasibility. The comprehensive use of positive behavioral support and peer-mediated strategies in teaching effectively shaped positive activity behaviors. It may also be related to the single session duration and overall cycle of the intervention. Most APA intervention studies for other ASD patients typically involve sessions of 30–45 minutes each, lasting no more than 6 months (35). In contrast, this study featured longer intervention duration and cycle (24 weeks, 80 minutes per session). Furthermore, the effectiveness of APA interventions is largely attributable to their design’s high alignment with the ICF-CY framework. The ICF-CY system comprehensively assesses each child with ASD across functional domains of body functions, activities and participation, and environmental interactions, establishing personalized plans based on these evaluations. Throughout APA interventions, children’s progress is dynamically monitored, with activity content adjusted promptly to ensure the continuity and efficacy of the intervention. The intervention goals are directly aimed at improving function, enhancing activity capacity, and promoting participation as well, rather than merely teaching motor skills.

Difficulties with social interaction are one of the core symptoms of children with ASD, often leading to social isolation and maladaptive behaviors (36). Targeted physical activity interventions can facilitate acquisition of communication skills, comprehension of social norms, and gradual social integration (9). Social adaptive ability serves as a bridge for children with ASD to connect with the external world (37). This study found that the ICF-CY-based APA intervention effectively improved independent function, cognitive function, and social self-control in children with ASD, thereby enhancing their social adaptive behaviors. Consistent with previous studies, comprehensive APA programs appear more conducive to improving these behaviors compared to single-form motor interventions. The improvement may be attributed to enhanced multisensory stimulation, including vestibular, proprioceptive, and tactile input. Promoting sensory integration and modulation, reducing social avoidance triggered by sensory hypersensitivity, and effectively facilitating social development in children with ASD (38). The systematic review and meta-analysis by Howells et al. (39) found that structured adaptive group exercise can significantly enhance motor skills and improve social functioning and communication in children with ASD. Within APA activities, appropriately challenging tasks, opportunities for autonomous choice, and peer support help fulfill children’s basic psychological needs, thereby fostering intrinsic motivation for social participation (40). Furthermore, modifications to the environment can positively influence the social development of children with ASD (41). In this APA program, instructors used verbal instructions, demonstrations, and whistle cues to assist children with ASD in completing activities. The adaptation of equipment and the requirement for children to collaboratively move apparatus also helped alter their relatively confined and monotonous school environment. The 24-week structured APA sessions, regular in timing and location, along with ongoing verbal stimulation and routines such as responding to roll calls, enabled children with ASD to consistently achieve phased motor goals. This process enhanced their trust in the environment and self-confidence, thereby boosting self-efficacy. Such positive psychological gains may generalize to other learning and life contexts, promoting the transfer and improvement of adaptive behaviors. In summary, the multi-faceted stimulation provided by APA positively influenced the social adaptive behaviors of children with ASD.

The APA program serves not only as an effective non-pharmacological intervention but also as a comprehensive approach to enhance physical activity participation and social interaction in children with ASD (17). This study demonstrates that the ICF-CY-based APA program yields significant intervention outcomes. By developing a structured and adaptable APA intervention model tailored to the physical and mental development of children with ASD, it provides a novel pathway for motor rehabilitation approaches in this population. Routine extracurricular physical activity sessions focus on functional domain of body functions recovery and basic motor skill training (42), prescribing intervention content based on disability categories and emphasizing short-term functional goals (43). However, it suffers from limitations such as neglecting environmental factors and individual differences, relying solely on single physical indicators for assessment, and lacking long-term planning for social participation (44). In contrast, the APA program under the ICF-CY framework is grounded in the biopsychosocial model. It begins with a comprehensive assessment of bodily functions, activity and participation levels, and environmental factors in children with ASD, leading to targeted APA intervention goals such as motor competence, activity engagement, health behaviors, and adaptation and empowerment. To achieve these goals, the program integrates a variety of strategies, including real-life scenario simulation, small-group cooperative games, team competitions, and guided education—to promote activity participation and social interaction. Light assistive equipment for daily living and communication is incorporated, while diverse interactive elements such as physical movement, verbal exchange, and eye contact are embedded within APA activities. This consistently supportive, encouraging, and actively engaging atmosphere is an essential factor throughout the long-term rehabilitation process for children with ASD.

## 5 Conclusions

The APA program, designed based on the ICF-CY theoretical framework, has been shown to effectively reduce SB and increase LPA, MVPA in children with ASD, while also enhancing their adaptive behavior levels. This comprehensive adapted physical activity model demonstrates feasibility and provides an empirical basis for further clinical research in autism rehabilitation. Moving forward, the ICF-CY-based APA program should be integrated into the comprehensive intervention and support systems for children with ASD, serving as a key practical approach to improving their health outcomes and promoting social inclusion.

## 6 Strengths and limitations

The strength of this study lies in its comprehensive and systematic assessment of children with ASD across the functional domain of body functions, activities and participation, and environmental factors, guided by the ICF-CY theoretical framework. Based on the assessment results, intervention goals were established and personalized APA plans were tailored, fully considering their physical and mental characteristics, individual differences, and environmental factors. This ensured the scientific rigor and specificity of the interventions.

This study has several limitations (1). It included only male participants. Although this aided in controlling sample homogeneity, it limits the generalizability of the findings. Given that gender may influence both participation in physical activity and adaptive behavior outcomes, we acknowledge that this design constitutes a potential source of bias. Future studies should include female participants to verify the generalizability of the results (2). The influence of subjective reporting factors on questionnaire results and wearer comfort issues, particularly for sensory-sensitive children with ASD, which may introduce bias in objective data. Future studies should incorporate more advanced, less intrusive monitoring devices with improved comfort, expand sample size and diversity, and enhance data reliability and generalizability. Future efforts should place greater emphasis on the role of the family environment, encouraging parental involvement in structured sessions and the creation of practice opportunities at home to promote skill generalization and support broader social integration and development.

## Data Availability

The original contributions presented in the study are included in the article/supplementary material. Further inquiries can be directed to the corresponding author.
